# The 5^th^ anniversary of “*Patient Safety in Surgery*” – from the Journal’s origin to its future vision

**DOI:** 10.1186/1754-9493-6-24

**Published:** 2012-10-18

**Authors:** Philip F Stahel, Wade R Smith, Dieter Hahnloser, Giuseppe Nigri, Cyril Mauffrey, Pierre-Alain Clavien

**Affiliations:** 1 Department of Orthopaedic Surgery, Denver Health Medical Center, University of Colorado, School of Medicine, Denver, CO, 80204, USA; 2 Department of Orthopaedic Surgery, Swedish Medical Center, Englewood, CO, 80113, USA; 3 Department of Visceral Surgery, University Hospital Lausanne (CHUV), CH-1011, Lausanne, Switzerland; 4 Department of Surgery, Sapienza University of Rome St. Andrea Hospital, 00189, Rome, Italy; 5 Department of Surgery, University Hospital Zurich, CH-8091, Zurich, Switzerland; 6 Department of Orthopaedics and Department of Neurosurgery, University of Colorado, School of Medicine, Denver Health Medical Center, 777 Bannock Street, Denver, CO, 80204, USA

## Inception of a new journal

“*Patient Safety in Surgery*” (PSS) was launched on November 7, 2007, as the first and only open-access, peer-reviewed, PubMed-cited online journal in the field of surgical patient safety [1]. Five years later, PSS remains the sole journal devoted to patient safety issues in surgery. The conception of the *Journal*’s mission originated in the summer of 2006 with a group of surgeon colleagues brain-storming about the meaning of the dogma that “*good judgment comes from experience which comes from poor judgment*”. During our routine weekly morbidity and mortality conferences in Denver and Zurich, we regularly unraveled severe complications generated by younger colleagues on their “learning curve”. Unfortunately, the patient is the one who eventually pays the price for the individual surgeon’s experience. We speculated about new options for sharing root causes of preventable incidents and complications, in order to avoid similar events to re-occur in a different patient in a different hospital. This fruitful debate led to the brainchild of creating a new international “forum” for exchanging case scenarios of specific surgical complications. This forum should be easily accessible, and include discussion of root causes, preventability, and action items needed for resolution and prevention of the future re-occurrence of identical, or similar, adverse events. Our debate also scrutinized the tendency of most standard print journals to publish positive data exclusively, with little room for negative results and reports on surgical failures and poor patient outcomes [2]. In a united consensus, we reasoned that the best option for creating a new forum of unrestricted reporting and debate on quality of care issues in the perioperative setting would be to start our own journal. The enthusiasm of the successive weeks let to the design of the mission statement for PSS (http://www.pssjournal.com/about). We further brain-stormed about the most suitable and representative project title for the new journal, and came up with some of the following tentative suggestions:

● “*Journal of Poor Judgment in Surgical Decision*-*Making*”

● “*Journal of Surgical Errors and Preventable Complications*”

● “*Journal of Surgical Complications*”

● “*Journal of Preventable Complications and Patient Safety*”

Ultimately, we decided to omit any negative aspect in the title, related to terms such as “errors” and “complications”, and instead to focus on an exclusively positive message in the final title, *Patient Safety in Surgery*[1]. With strong support from the publisher, BioMedCentral (BMC), the new journal was successfully launched on November 7, 2007, accompanied by the first two peer-reviewed articles.

## The mission

As outlined on the PSS homepage (http://www.pssjournal.com), the *Journal*’s mission is to increase the safety and quality of care for patients undergoing surgical procedures in all fields of surgery. The *Journal* was designed to complement the more than 200 traditional surgical journals by filling an essential void, through providing a forum for discussion, analysis, and work-up of system and process failures, technical complications, medical errors, and other adverse events in the management of surgical patients in the perioperative setting. This scientific forum was created to lower the threshold for reporting adverse events in all fields of surgery, with the long-term goal of increasing the safety and quality of care for patients undergoing surgical procedures. Ultimately, health care providers from around the world need to be able to safely and openly report anything that does not go well for their patients. As surgeons, we are the only group of people on earth whom other humans give formal consent to render them unconscious and open their bodies with a knife. Thus, we have the highest onus to leave no stone unturned in our quest to do the “right thing” for our patients. We strongly consider public reporting of medical errors and surgical complications an ethical responsibility of our profession. Therefore, we will continue to strive to offer PSS as a vehicle of transparency, trust, and credibility for the public who has a right to know the truth about the quality and safety of surgical care provided around the globe.

## The first 5 years: a spectacular start

**Figure 1:**
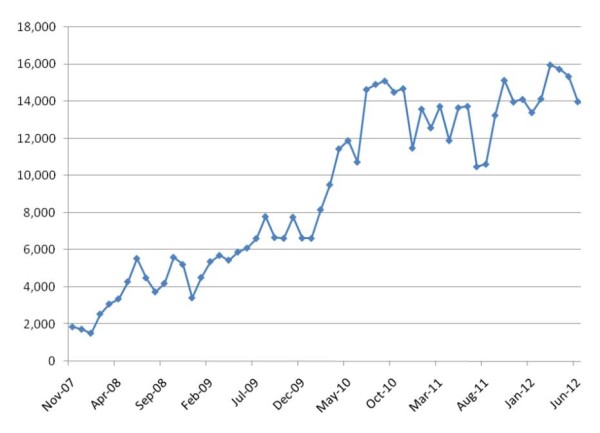
**Article accesses to *Patient Safety in Surgery.*** The graph shows the growing number of accesses to PSS articles, from the time of the *Journal*’s launch in November 2007, until June 2012. The data reflect access statistics to the PSS webpage exclusively, and do not include additional sources of access, including PubMed and other portals and article repositories.

During the first 5 years, PSS had a spectacular beginning, a notion which is supported by the following actual statistics:

● The readers’ access to papers published on the PSS website (http://www.pssjournal.com) has increased from less than 2,000 hits in 2007, to up to 16,000 accesses per month in 2012 (Figure 1).

● The top-25 most accessed articles have been viewed through the PSS website more than 500,000 times until present (http://www.pssjournal.com/mostviewed/alltime).

**Figure 2:**
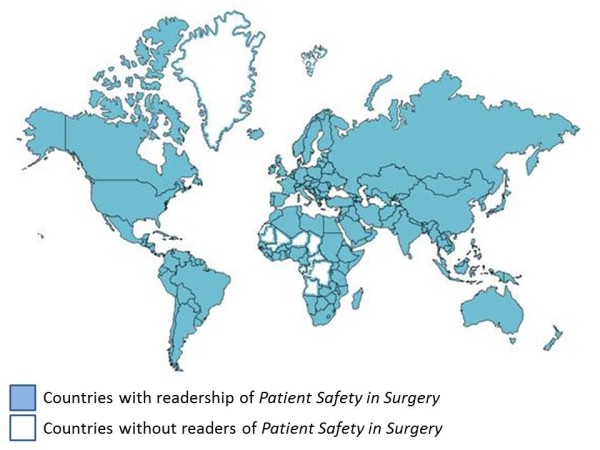
**Global readership of *Patient Safety in Surgery.*** All countries with previous access to articles published in PSS are marked in blue background. The few unmarked countries do not have a history of access to the *Journal*. These selected states include Greenland, Turkmenistan, Tajikistan, and some countries in West Africa and Central Africa.

● The *Journal* is supported by an internationally renowned editorial board of 59 editors from 15 different countries (http://www.pssjournal.com/edboard), and is read online in more than 180 countries around the world (Figure 2).

● PSS has an “unofficial” impact factor of 1.19 (http://www.pssjournal.com). This impact factor is calculated by the identical formula as for the *Journal Citation Reports* (*JCR*®) and thus reflects a “real” number for the journal’s impact factor, albeit not (yet) officially sanctioned by Thomson Reuters [3].

**Figure 3:**
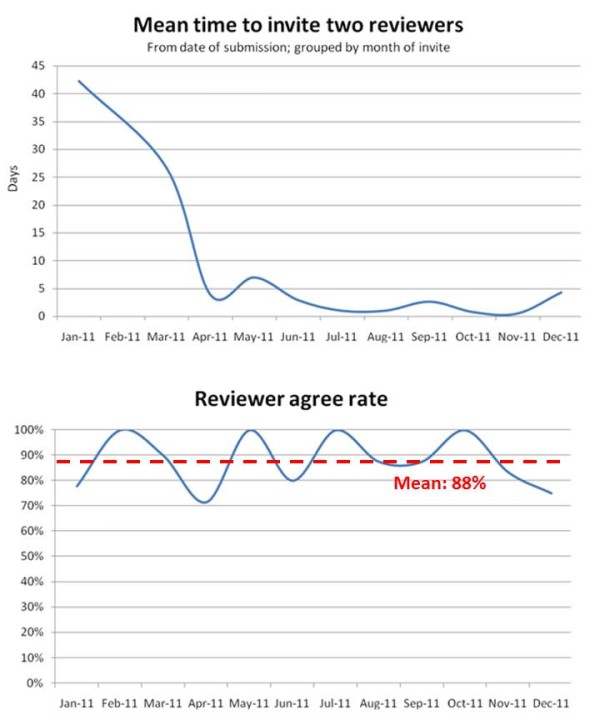
**Reviewer invitation time(upper panel)and invite acceptance rate** (**lower panel**) **in 2011.** The mean time to assign two individual referees to evaluate a submitted manuscript has been drastically reduced in 2011, from originally more than 40 days, to currently less than 5 days (from day of submission). Furthermore, an average of 88% of all invited reviewers accepted their assignment in 2011. This efficient recruitment of qualified referees allows for the fast-track management of submitted manuscripts, and guarantees short turn-around times until a first editorial decision is made.`

Of all manuscripts published in PSS from 2010–2011, the most frequently cited article has been cited 15 times in other journals [4], and the most accessed paper has been viewed through the *Journal*’*s* webpage more than 16,000 times [5]. Overall, more than 140 articles have been published in PSS until present, with an average rejection rate of 20%. The *Journal* continues to publish two editorials per calendar year which address controversial topics in the field (/http://www.pssjournal.com/content?articleTypes=Editorial). The average time from manuscript submission to a first editorial decision is currently one month (30 days), and the time to a final decision lies around two months from initial submission (40–80 days). The “key” to a successful fast-track management of submitted manuscript lies in the timely appointment of suitable referees, and the reliance on their timely acceptance and commitment to returning their reviews within 14 days. Overall, an average of 88% of all invited referees accept their assignment within less than 5 days from manuscript submission, a statistic which speaks for the quality of the initial reviewer selection by the managing editors (Figure 3).

A meta-analysis of post-publication surveys from authors who have previously published in the *Journal* reveals the following primary reasons for submitting their work to PSS:

(1) Journal scope / Good prior experience

(2) Journal reputation and profile

(3) Fast peer review / Speed of publication

Overall, within just 5 years of the initial launch, PSS has been able to solidify its early reputation as an internationally respected journal in the important field of patient safety in the perioperative setting.

## Challenges and road bumps

The design and implementation of PSS as a new journal in its field was accompanied by multiple challenges and hurdles. As an anecdotal example, many friends and colleagues (as well as the publisher’s legal advisers!) discouraged the founding editors from introducing an article category on “case reports”. The underlying argument was that “*only a fool will agree to publish a case report on preventable complications which lead to poor patient outcomes*”. This notion is based on the rationale that such a document would provide a written testimony (and admission of guilt) which could be used in court against the individual medical practitioner in case of a malpractice claim or lawsuit. The ultimate resolution consisted of introducing a mandatory request for submitting authors to provide a written consent from patients or their legal guardians for any manuscript which provides information on specific identifiable individual patient scenarios (http://www.pssjournal.com/authors/instructions).

Strikingly, we were astonished by the unexpected high submission rate of case reports on surgical complications, preventable sentinel events, and “never events”, starting from the first weeks of the journal’s launch [6,7], until the present day [8,9]. This impressive fact supports the notion that health care providers all over the globe appear to strive to publicly report, analyze, and discuss root causes and preventive measures of adverse events which lead to unnecessary patient harm, in order to provide more transparency to other health care providers, and to the public. Indeed, until present, PSS published a total of 50 case reports on individual complications and medical errors, and the manuscript submissions in the “case report” category keep coming in.

Beyond a doubt, the main barrier which deters authors from submitting their work to PSS (and for that matter to open access journals in general) is represented by the extremely high publication fees. For journals published by BMC, these so-called “article processing charges” (APCs) – which have to be carried by the author – range from $1,600 to $1,900 per article (http://www.biomedcentral.com/about/apcfaq). A recent commentary published in *Science* discussed the findings of a large-scale survey on the perceived role of open access online journals among 50,000 researchers [10]. While 89% of all respondents expressed their support for open access publishing in general, they admitted to publish only about 10% of their own research in open access journals [10]. The two main reasons stated for the poor submission rate were high publication fees (40%) and the apparent lack of high-quality open access journals in the respondents’ field of interest (30%) [10].

And here lies the conundrum of open access publishing: Why would a hypothetical author submit high-quality research to an open access journal with low reputation and no (or low) impact factor, which comes at a price of up to $2,000 publication costs, instead of targeting a prestigious high-impact print journal, free of charge? The answer is intuitive.

## Incentives to publish in PSS

PSS has seen a tremendous start in its first 5 years, and we have successfully resolved some of the initial “childhood diseases” of any new journal. In order to overcome the financial impediment for submission of high-quality articles imposed by the high processing fees, we were recently able to obtain independent grant support for coverage of the APC fees for 30 articles submitted by outside researchers. Pre-submission inquiries for qualification of a waiver for processing fees should be submitted to the editorial board (philip.stahel@dhha.org). Requests will be screened by the managing editors based on objective metrics related to the manuscript’s scientific quality. An anecdotal example of a manuscript which was actively commissioned for PSS, with the authors’ APC fees being waived through extramural grant support, shows that this paper is currently ranked as the #1 most viewed article in the *Journal* of all times, with more than 23,000 accesses until present [11]. This example supports the argument that high-quality submissions are difficult to commission in presence of the financial “APC barrier”.

Undoubtedly, the open access modality of publishing in PSS provides unprecedented advantages compared to standard print journals. The elimination of financial barriers related to individual or institutional journal subscriptions allows for global and unrestricted free access to all published articles. This proactive modality likely represents the underlying reason for a new journal, such as PSS, being read in more than 180 countries around the world (Figure 2). In addition, submitting authors from developing countries can request a formal APC waiver by the publisher, which is usually granted within a few days of the inquiry.

The following aspects provide some irrefutable incentives for publishing in PSS:

● All articles published in PSS are free to read, copy, distribute, and re-publish in parts or in entirety (with attribution of the original source).

● Authors retain the full unrestricted copyright on the entire article. This allows for replicating data and figures in future publications (e.g. review articles or book chapters) without the need for requesting a formal copyright release by the publisher, as long as the original source is adequately cited.

● There is no limit to the length of an individual article, including the number of tables and figures.

● There are no extra charges for publishing an unrestricted amount of high-quality color figures.

● Movie clips can be embedded for instructional purposes at no extra cost, through embedded links in the manuscript (see example: [12]).

● The fast-track publication process allows for short turn-around times of submitted manuscripts and publication within about 2 months of initial submission. All articles are cited in PubMed within less than one week of provisional publication.

● All articles published in PSS are archived in public repositories, including PubMed Central, in compliance with the NIH Public Access Policy.

## The next 5-year vision

The first international editorial board meeting for PSS took place on October 3, 2012, at the Clinical Congress of the *American College of Surgeons* (ACS) in Chicago, IL. During this meeting, we discussed current challenges and outlined the future vision for the *Journal* in the next 5 years. One priority is to take PSS from its first unofficial impact factor of 1.19 to an official impact factor in the *Journal Citation Reports* (*JCR*®). This goal precludes the prospect of being tracked by Thomson Reuters for an official impact factor. Indeed, selected online open access journals have accomplished the task of “squaring the circle” by achieving high impact factors which are competitive with standard print journals in their respective field. Impressively, one representative open access journal from the “Public Library of Science”, *PLoS Biology*, has reached an impact factor of 11.45, which ranks the journal #1 in the *JCR*® category of ‘Biology’ in 2011. Additional examples of selected open access online journals with noteworthy impact factors are listed below (year of launch and current impact factor in parentheses):

● *Journal of Hematology* &*Oncology* (2008; IF 3.99)

● *PLoS ONE* (2006; IF 4.09)

● *Molecular Neurodegeneration* (2006; IF 4.28)

● *Journal of Neuroinflammation* (2004; IF 3.83)

● *Retrovirology* (2004; IF 6.47)

● *BMC Biology* (2003; IF 5.75)

● *PLoS Biology* (2003; IF 11.45)

● *Genome Biology* (2000; IF 9.04)

● *Breast Cancer Research* (1999; IF 5.25)

These selected open access journals serve as pioneer role models with regard to the gradual transition from the “classic” entity of print publication to the future model of open access publishing of high quality science in high impact online journals. Clearly, the impact factor alone does not guarantee the quality of submitted papers, but rather represents a surrogate marker for the scientific renown of a journal in the international community. However, in this day and age of restricted grant funding opportunities, a journal with a (high) impact factor is more likely to be targeted by better quality submissions.

Another important and challenging goal for the next 5 years includes the plan to inaugurate an “*International Society for Patient Safety in Surgery*”. This new society will further enhance the current global patient safety initiative aimed at increasing transparency about the quality of medical care to the public, who remains the ultimate stakeholder [13]. The obligation for us, as physicians and surgeons, lies in sustaining our public credibility by full and honest disclosure and reporting of medical errors and surgical complications [14].

Ultimately, PSS strives to position itself as a landmark journal in the field of patient safety in the perioperative setting, with a global renown for publishing high-quality science in this important field. There is a lot of work ahead of us. We would like to thank our readership, submitting authors, peer reviewers, and our editorial board for their loyalty in the past 5 years, and for their continuing support of the journal’s mission in the future.

## Competing interests

The authors are all members of the *Journal*’s editorial board (http://www.pssjournal.com/edboard/). They declare no other conflicts of interest.

## Authors’ contributions

PFS, PAC, DH and GN designed this editorial. PFS wrote the first draft of the article. All authors contributed to revisions and approved the final version of the manuscript.
